# MiR-506 exerts antineoplastic effects on osteosarcoma cells via inhibition of the Skp2 oncoprotein

**DOI:** 10.18632/aging.202530

**Published:** 2021-02-17

**Authors:** Lu Ding, Rongxin Sun, Qi Yan, Chengwei Wang, Xiaoping Han, Yong Cui, Rong Li, Jiwen Liu

**Affiliations:** 1 Postdoctoral Research Center on Public Health and Preventive Medicine, Xinjiang Medical University, Xinjiang, China; 2 Fifth Affiliated Hospital, Xinjiang Medical University, Xinjiang, China; 3 Department of Orthopedics, Sixth Affiliated Hospital, Xinjiang Medical University, Xinjiang, China; 4 Department of Maternal, Child and Adolescent Health, College of Public Health, Xinjiang Medical University, Xinjiang, China; 5 Tumor Hospital Affiliated to Xinjiang Medical University, Xinjiang, China; 6 Postdoctoral Research Center on Clinical Medicine, First Affiliated Hospital, Xinjiang Medical University, Xinjiang, China

**Keywords:** osteosarcoma, miR-506, Skp2, cell proliferation, invasion, apoptosis

## Abstract

S-phase kinase-associated protein 2 (Skp2) performs oncogenic functions in cancers; however, how Skp2 is regulated post-transcriptionally is elusive in osteosarcoma. Therefore, we determined whether miR-506 could directly target Skp2 in osteosarcoma to perform its tumor suppressive functions. Here, we found that miR-506 mimics suppressed cell viability, induced apoptosis, and attenuated migration and invasion in osteosarcoma cells. Moreover, upregulation of Skp2 accelerated cell viability and motility and rescued the tumor suppressive effect of miR-506 in osteosarcoma cells. Moreover, downregulation of Skp2 inhibited cell viability and decreased cell motility, which enhanced the antitumor activity induced by miR-506 mimic transfection in osteosarcoma cells. Our western blotting results implied that miR-506 inhibited Skp2 expression and subsequently upregulated Foxo1 and p57 in OS cells. In summary, miR-506 performs an anticancer activity via directly targeting Skp2 in osteosarcoma cells, indicating that inactivation of Skp2 by miR-506 might be an alternative strategy for treating osteosarcoma.

## INTRODUCTION

Osteosarcoma is a malignant bone pleomorphic tumor in children and adolescents [1]. The chemotherapeutic agents for osteosarcoma often include methotrexate, doxorubicin, cisplatin and ifosfamide [2]. With advances in several therapeutic approaches, the 5-year survival rate of osteosarcoma patients is about 70% [3, 4]. Patients with metastatic osteosarcoma or recurrent tumors often have worse outcomes [5, 6]. Hence, the identification of new therapeutic markers is necessary to improve patient care and obtain a favorable outcome in osteosarcoma.

It is known that a family of noncoding RNA molecules, known as microRNAs (miRNAs), is critically involved in carcinogenesis and tumor progression [7, 8]. For example, miR-506 (also known as miR-506-3p) has been found to regulate oncogenesis in human cancers, including osteosarcoma [9]. One study showed that miR-506-3p suppressed proliferation and stimulated apoptosis via regulation of the Sirtuin 1 (SIRT1)/Akt/FOXO3a pathway in ovarian cancer cells [10]. Moreover, miR-506 was reported to control proliferation and apoptosis via regulation of the RhoA/Rho-associated protein kinase (ROCK) pathway in hepatocellular carcinoma cells [11]. Overexpression of miR-506 suppressed metastasis of gastric carcinoma cells by targeting zinc finger E-box-binding homeobox 2 (ZEB2) [12]. Similarly, miR-506 attenuated tumor growth and metastasis via inactivation of the Wnt/β-catenin pathway by inhibiting LIM homeobox 2 (LHX2) in nasopharyngeal carcinoma [13]. It has been reported that miR-506 expression levels are decreased in osteosarcoma specimens compared with paired normal bone tissues [14–16]. Upregulation of miR-506 suppressed cell growth and invasiveness by targeting Snail2 in osteosarcoma cells [14]. Similarly, promotion of miR-506 inhibited proliferation and enhanced apoptosis by targeting AEG-1 in osteosarcoma cells [15]. These reports indicate that miR-506 plays an essential role in osteosarcoma.

Skp2 is identified as an oncoprotein that exhibits tumor-promoting functions in malignancies [17]. It has been shown that Skp2 has a critical role in the regulation of cellular processes such as differentiation, proliferation, apoptotic death, motility and the cell cycle via the ubiquitination and degradation of Skp2 substrates [18, 19]. The substrates of Skp2 often are tumor suppressors including p21, E-cadherin, p27, p57, FOXO1 and p130 in human cancer cells [19]. Upregulation of Skp2 has been reported in a broad spectrum of human cancers, including breast, prostate, pancreatic cancer, and osteosarcoma [20–22]. For example, saurolactam, a natural agent from Saururus chinensis, suppressed proliferation and motility in osteosarcoma cells in part by targeting Skp2, indicating that Skp2 is involved in osteosarcoma progression [23]. In addition, 15,16-dihydrotanshinone I inhibited proliferation and migration and induced apoptosis partly by targeting Skp2 in osteosarcoma cells [24].

Our previous studies showed that inhibition of Skp2 suppressed cell viability, stimulated apoptosis and led to cell cycle arrest, and attenuated cell migrative ability in osteosarcoma cells, whereas overexpression of Skp2 exerted the opposite effects on osteosarcoma cells [25, 26]. Similarly, one group showed that suppression of Skp2 attenuated cell invasion and lung metastasis in osteosarcoma [27]. Moreover, we reported that Skp2 is involved in methotrexate-mediated resistance of osteosarcoma cells due to the induction of epithelial-mesenchymal transition [28]. Although Skp2 has a critical role in osteosarcoma, the regulatory mechanism of Skp2 is unclear. In the current study, we present a new regulatory mechanism of Skp2 in osteosarcoma cells. We reported that miR-506 reduced cell viability and motility and induced apoptosis by targeting Skp2 in osteosarcoma cells.

## RESULTS

### Overexpression of miR-506 reduces cell viability

**Figure 1:**
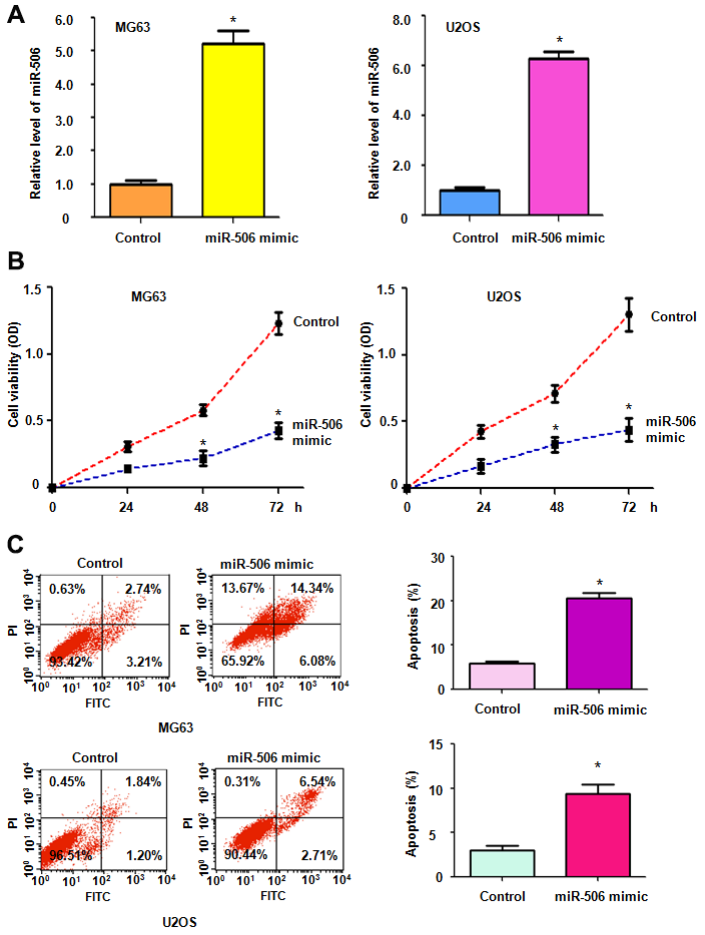
**Upregulation of miR-506 suppresses cell viability.** (**A**) The miR-506 level was evaluated by RT-PCR in osteosarcoma cells transfected with miR-506 mimics. *p < 0.05, compared to control. (**B**) Cell viability was detected by MTT method in MG63 and U2OS cells after miR-506 overexpression. (**C**) Left panel: Apoptosis was evaluated by flow cytometry in osteosarcoma cells after miR-506 mimic transfection. Right panel: Quantification of apoptosis results.

To dissect the function of miR-506 in osteosarcoma cells, we transfected miR-506 mimics into osteosarcoma cells. The data showed that miR-506 is highly expressed after miR-506 mimic treatments in both MG63 and U2OS cells (Figure 1A). To determine the effects of miR-506 mimics on cell viability, an MTT assay was conducted in osteosarcoma cells after transfection with miR-506 mimics for different days. We observed that miR-506 upregulation alleviated the viability of MG63 and U2OS cells (Figure 1B). Moreover, our MTT data indicated that miR-506 overexpression attenuated cell viability in a time-dependent manner in osteosarcoma cells (Figure 1B).

### Overexpression of miR-506 stimulates cell apoptosis

We further explored whether miR-506 affected apoptosis in osteosarcoma cells. Transfection of miR-506 mimics induced apoptosis in MG63 and U2OS cells (Figure 1C). The percentage of apoptotic cell death was elevated from 6% to 20% after the miR-506 mimic transfection in MG63 cells (Figure 1C). Similarly, apoptosis in U2OS cells was increased from 2.2% to 9.3% after the miR-506 mimic- treatment (Figure 1C). This finding indicated that miR-506 overexpression induced cell apoptosis, leading to cell viability inhibition.

### Overexpression of miR-506 retards cell motility

**Figure 2:**
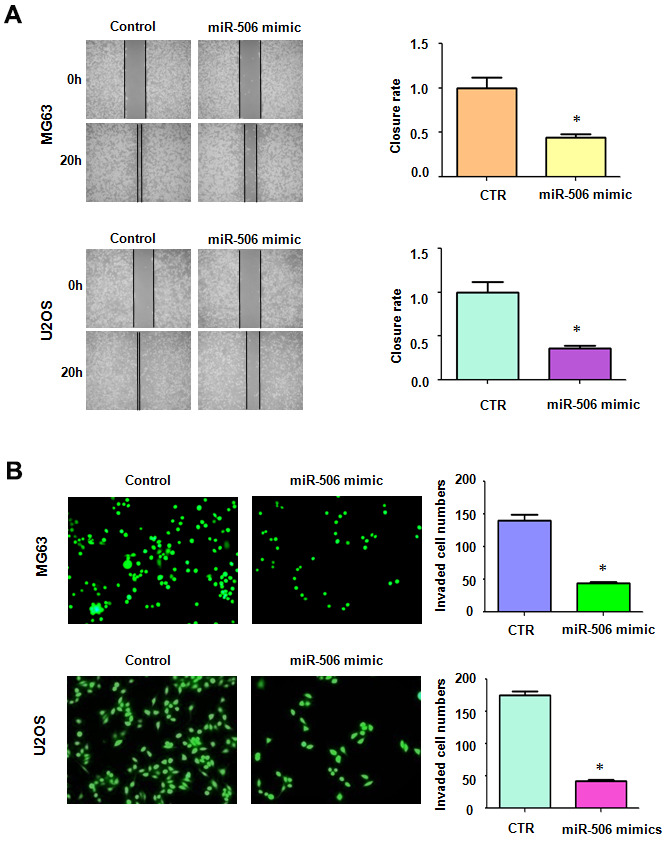
**Overexpression of miR-506 inhibited cell motility.** (**A**) Left: Migrative ability was evaluated by wound healing approach in osteosarcoma cells after miR-506 mimic treatment. Right: Quantitative results of migration. *p< 0.05, compared to control. (**B**) Left: Invasion was evaluated by Transwell assay in osteosarcoma cells treated with miR-506 mimics. Right: Quantitative results of invasion.

Next, we unraveled the effect of miR-506 on migrative ability and invasive activity of osteosarcoma cells. Our wound healing assay denoted that increased miR-506 retarded the migratory activity of osteosarcoma cells (Figure 2A). Moreover, miR-506 mimic transfection in osteosarcoma cells led to suppression of cell penetration through the Matrigel-covered membrane (Figure 2B). Overall, miR-506 participates in the regulation of migratory and invasive activities of osteosarcoma cells.

### Skp2 is a target of miR-506

**Figure 3:**
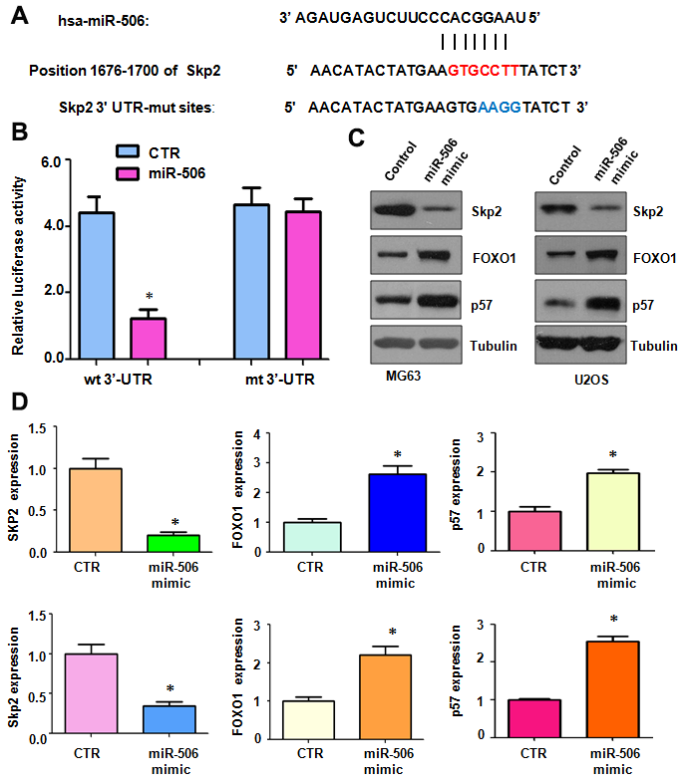
**Skp2 is a target of miR-506.** (**A**) The interaction between miR-506 and the Skp2 3’UTR is shown. Mutation of the putative miR-506–binding site on the Skp2 3’UTR is presented. (**B**) A luciferase reporter assay was carried out to test the binding of miR-506 to the Skp2 3’-UTR. CTR: Control. WT: wild type; Mt: mutation. (**C**) Western blotting detection of the protein level of Skp2 and its targets in osteosarcoma cells transfected with miR-506 mimics. (**D**) Quantitative results of (**C**). *p < 0.05, compared to control.

To identify a miR-506 target, we used several miRNA prediction tools, including miRTar, TargetScan, miRDB, and miRanda, to predict the downstream target of miR-506. Skp2 was predicted as a potential target of miR-506. We identified one binding site between the Skp2 3’-UTR sequence and the miR-506 sequence (Figure 3A). To confirm the interaction between miR-506 and the Skp2 3’-UTR, we conducted a dual luciferase reporter assay in U2OS cells. Our results demonstrated that miR-506 mimics reduced the luciferase activity of the wild-type Skp2 3’UTR, but not the mutant-type Skp2 3’UTR reporter (Figure 3B). Furthermore, our data illustrated that miR-506 upregulation diminished the Skp2 expression in MG63 and U2OS cells (Figure 3C, 3D). Accordingly, miR-506 mimics reduced Skp2 expression and subsequently promoted the expression of downstream targets, FOXO1 and p57, in osteosarcoma cells (Figure 3C, 3D). These results suggest that Skp2 might be a potential target of miR-506.

### Skp2 overexpression abrogates miR-506-induced viability reduction and apoptosis

**Figure 4:**
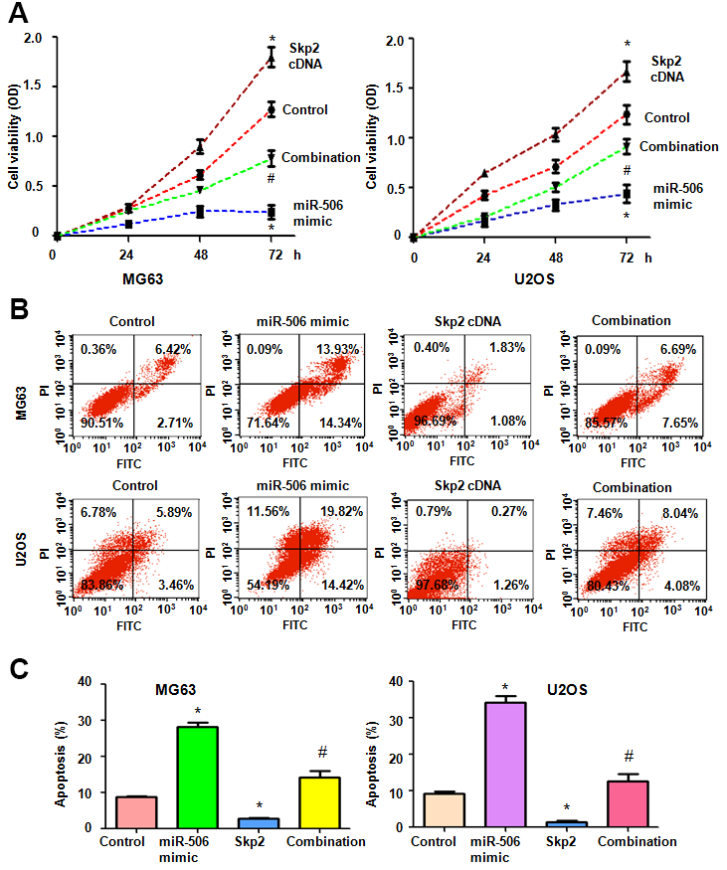
**Skp2 overexpression abrogates miR-506-mediated cell viability inhibition and apoptosis induction.** (**A**) Viability was detected by MTT in osteosarcoma cells after miR-506 overexpression and Skp2 upregulation. *p < 0.05 vs control, ^#^P<0.05 vs miR-506 mimics or Skp2 cDNA transfection. (**B**) Apoptosis was examined by flow cytometry in osteosarcoma cells after miR-506 mimic transfection and Skp2 upregulation. (**C**) Quantification of apoptosis results.

To ascertain whether miR-506 utilizes its antineoplastic activity via inhibition of Skp2 in osteosarcoma cells, we transfected Skp2 plasmid into cells and then added miR-506 mimics. The viability of osteosarcoma cells after Skp2 plasmid and miR-506 mimic transfections was evaluated by MTT assay. The MTT data showed that upregulation of Skp2 promoted cell viability, while miR-506 mimics diminished viability of osteosarcoma cells (Figure 4A). Skp2 overexpression rescued the miR-506-induced viability suppression in MG63 and U2OS cells (Figure 4A). Apoptosis was measured in osteosarcoma cells after Skp2 plasmid and miR-506 mimic transfections. Skp2 upregulation inhibited apoptosis, while miR-506 mimics triggered apoptosis in osteosarcoma cells (Figure 4B, 4C). Strikingly, upregulation of Skp2 abrogated the miR-506-mediated apoptosis induction (Figure 4B, 4C).

### Skp2 overexpression rescues miR-506-induced inhibition of motility

**Figure 5:**
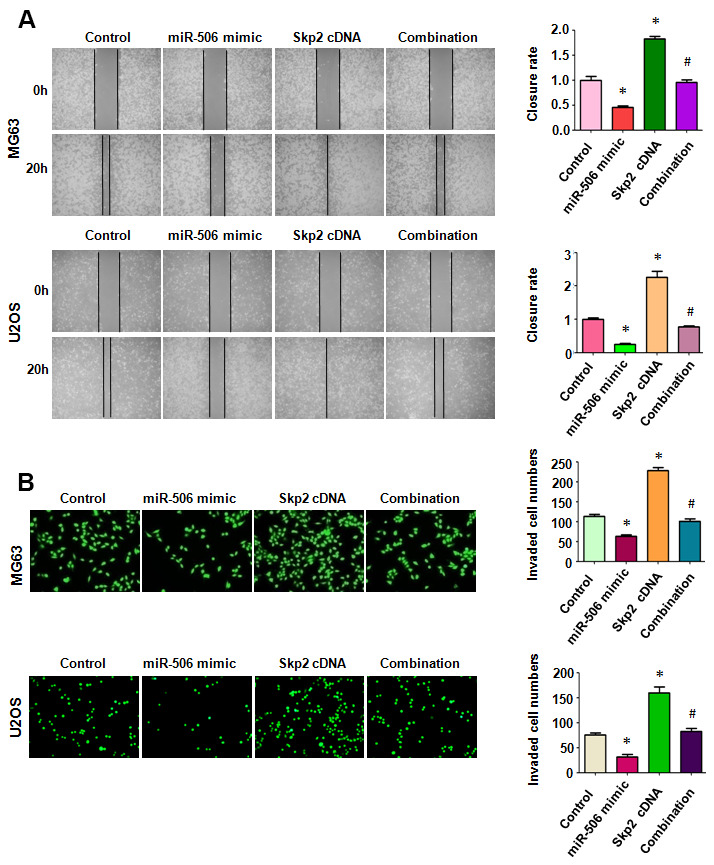
**Skp2 overexpression rescues miR-506-induced inhibition of motility.** (**A**) Left: Wound healing assay was done in osteosarcoma cells after miR-506 mimic transfection and Skp2 upregulation. Right: Quantitative results of migration. *p < 0.05 vs control, ^#^P<0.05 vs miR-506 mimics or Skp2 cDNA transfection. (**B**) Left: Invasion was examined by Transwell assay in osteosarcoma cells after miR-506 mimic transfection and Skp2 upregulation. Right: Quantitative results of invasion.

To define whether miR-506 performed anti-motility effects via regulation of Skp2 in osteosarcoma cells, we measured the migratory activity of osteosarcoma cells after miR-506 mimic transfection in combination with Skp2 plasmid transfection. We found that Skp2 upregulation increased cell migration, while miR-506 overexpression retarded the migratory activity in osteosarcoma cells (Figure 5A). Overexpression of Skp2 attenuated miR-506-triggered inhibition of migration in MG63 and U2OS cells (Figure 5A). Moreover, Transwell chamber invasion assay data showed that Skp2 plasmid transfection increased cell invasiveness and that miR-506 upregulation retarded cell invasion in osteosarcoma cells (Figure 5B). In addition, high expression of Skp2 blocked miR-506-mediated suppression of invasion of osteosarcoma cells (Figure 5B).

### Skp2 overexpression attenuates miR-506-induced upregulation of FOXO1 and p57

**Figure 6:**
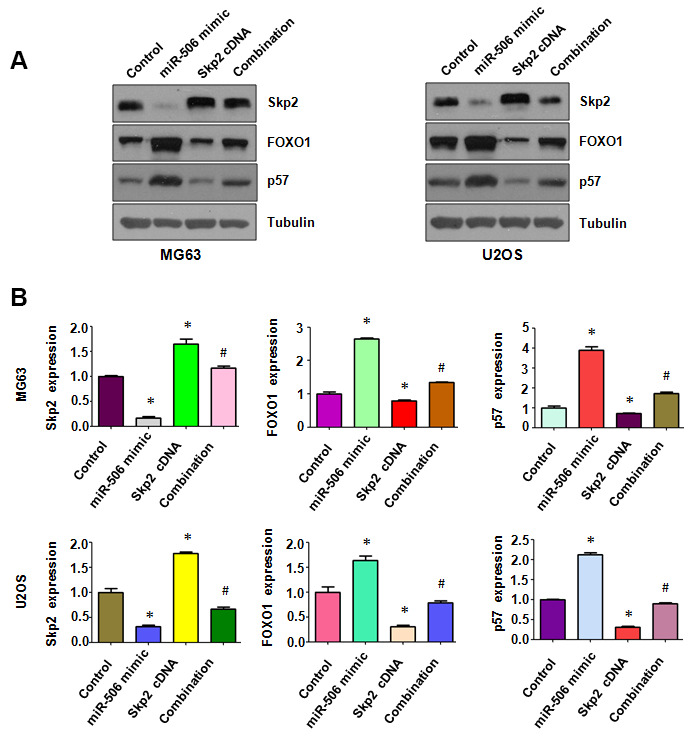
**Skp2 overexpression attenuates miR-506-induced upregulation of FOXO1 and p57.** (**A**) Western blotting detection of the protein level of Skp2 and its targets in osteosarcoma cells after miR-506 mimic transfection and Skp2 upregulation. (**B**) Quantitative results of (**A**). *p < 0.05 vs control, ^#^p < 0.05 vs miR-506 mimics or Skp2 cDNA transfection.

Skp2 expression level in osteosarcoma cells after miR-506 mimics plus Skp2 plasmid cotransfection was determined by immunoblotting. We observed that Skp2 cDNA plasmid transfection promoted the expression of Skp2 in osteosarcoma cells (Figure 6A, 6B). Moreover, miR-506 overexpression inhibited Skp2 expression in osteosarcoma cells, which was rescued by Skp2 overexpression (Figure 6A, 6B). FOXO1 and p57 expression was inhibited by Skp2 upregulation (Figure 6A, 6B). Overexpression of miR-506 upregulated the expression of p57 and FOXO1, which was abrogated by miR-506 mimic transfection in osteosarcoma cells (Figure 6A, 6B).

### Skp2 downregulation enhances miR-506-mediated antitumor activity

**Figure 7:**
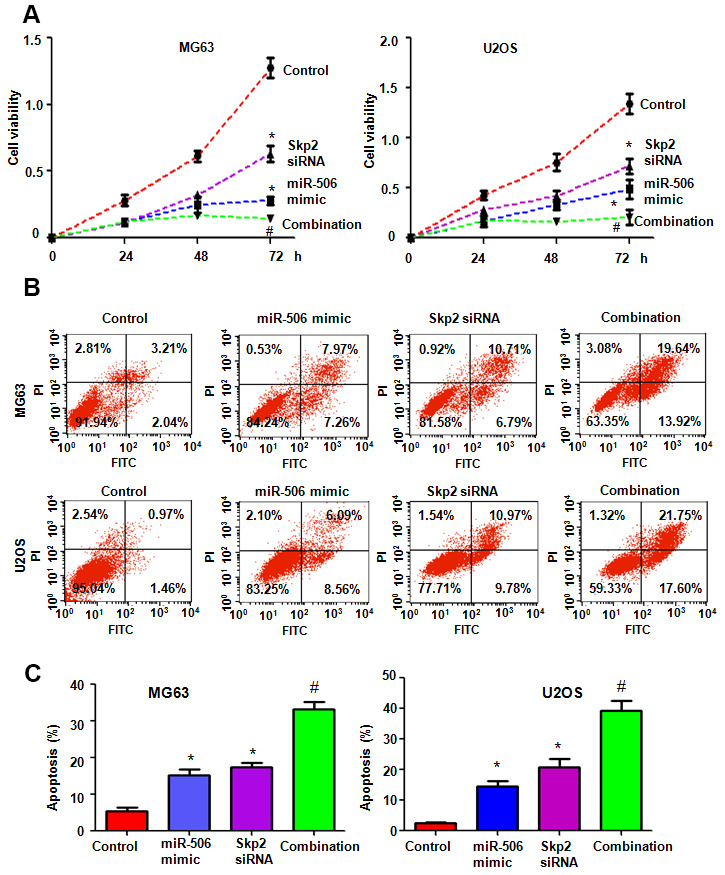
**Skp2 downregulation enhances miR-506-mediated viability inhibition and apoptosis induction.** (**A**) Viability was examined by MTT assay in cells after miR-506 overexpression and Skp2 downregulation. *p < 0.05 vs control, ^#^P<0.05 vs miR-506 mimics or Skp2 siRNA transfection. (**B**) Apoptosis was examined by flow cytometry in osteosarcoma cells after miR-506 mimic transfection and Skp2 downregulation. (**C**) Quantification of apoptosis results.

**Figure 8:**
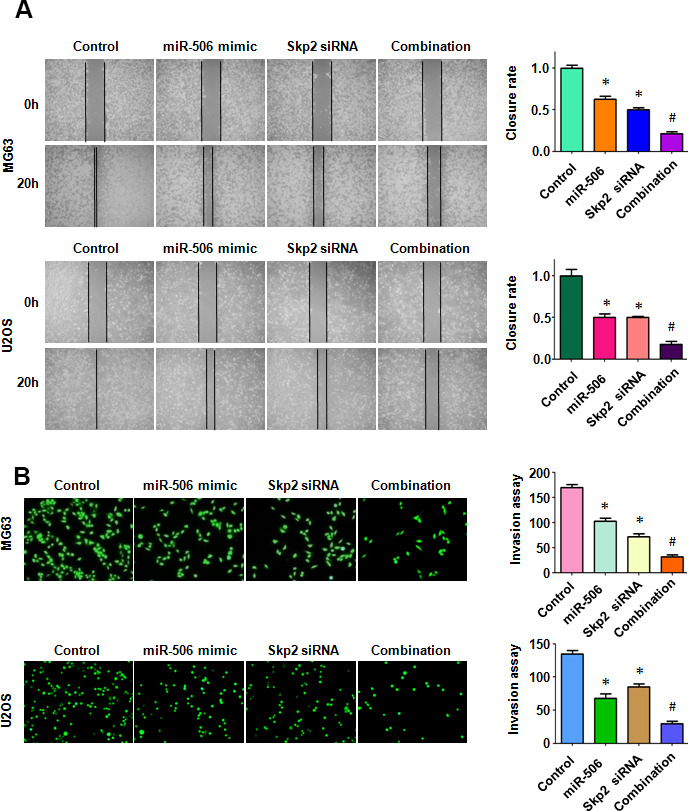
**Skp2 downregulation enhances miR-506-induced inhibition of motility.** (**A**) Left: Wound healing assay was conducted in osteosarcoma cells after miR-506 mimic transfection and Skp2 downregulation. Right: Quantitative results of migration. *p < 0.05 vs control, ^#^P<0.05 vs miR-506 mimics or Skp2 siRNA transfection. (**B**) Left: Invasion ability was examined by Transwell assay in osteosarcoma cells after miR-506 mimic transfection and Skp2 downregulation. Right: Quantitative results of invasion.

**Figure 9:**
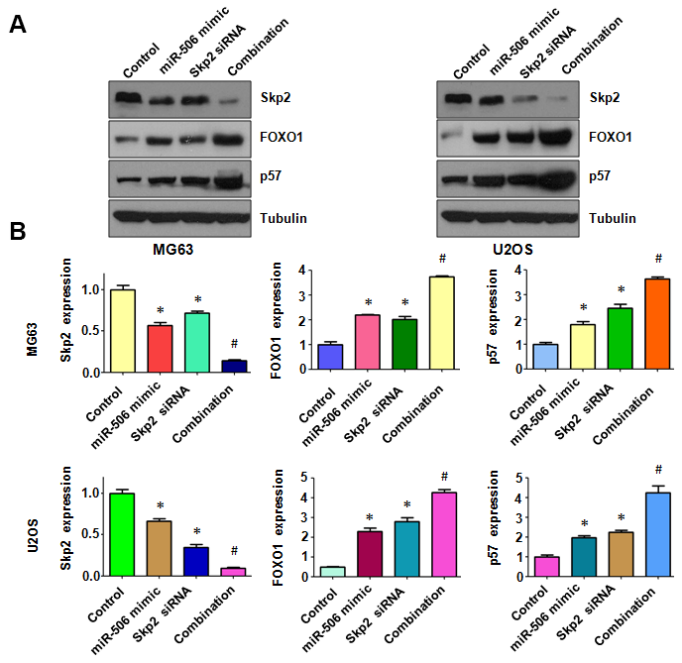
**Skp2 downregulation enhances miR-506-induced upregulation of FOXO1 and p57.** (**A**) Immunoblotting was done to evaluate the protein level of Skp2 and its targets in osteosarcoma cells after miR-506 mimic transfection and Skp2 downregulation. (**B**) Quantitative results of (**A**). *p < 0.05 vs control, ^#^p < 0.05 vs miR-506 mimics or Skp2 siRNA transfection.

To dissect whether miR-506 performs antitumor activity by targeting Skp2 in osteosarcoma cells, we transfected Skp2 siRNA and miR-506 mimics into MG63 and U2OS cells. Our MTT assay results demonstrated that Skp2 downregulation attenuated the viability of osteosarcoma cells and enhanced miR-506-induced inhibition of cell viability (Figure 7A). Moreover, Skp2 siRNA transfection led to increased apoptosis and promoted miR-506-induced apoptosis in osteosarcoma cells (Figure 7B, 7C). Furthermore, downregulation of Skp2 moderated motility of osteosarcoma cells (Figure 8A, 8B). Notably, Skp2 reduction by siRNA transfection facilitated the retardation of motility by miR-506 mimic transfection (Figure 8A, 8B). Skp2 expression was reduced at a lower level in osteosarcoma cells treated with both Skp2 siRNA and miR-506 mimics compared with Skp2 siRNA transfection alone or miR-506 mimic transfection alone (Figure 9A, 9B). In line with this result, FOXO1 and p57 expressions were increased at a larger level in the Skp2 siRNA transfection plus miR-506 mimic cotransfection group compared with Skp2 downregulation or miR-506 overexpression alone (Figure 9A, 9B).

## DISCUSSION

In this study, miR-506 overexpression suppressed cell viability, triggered apoptosis, and reduced migration and invasion of osteosarcoma cells. Moreover, upregulation of Skp2 abrogated the antitumor function of miR-506 in osteosarcoma cells. Similarly, downregulation of Skp2 enhanced the antitumor activity induced by miR-506 overexpression in osteosarcoma cells. This study demonstrated that suppression of Skp2 via upregulation of miR-506 might be a potential approach for osteosarcoma therapy.

Skp2 can be regulated by multiple miRNAs in human tumors. For instance, that miR-7 induced G1 to S phase transition through reduction of Skp2 and Psme3 in CHO cells [29]. The miR-30 family targeted Skp2 in the premetastatic phase in the lungs of B16 tumor-bearing mice, leading to inhibition of pulmonary vascular hyperpermeability [30]. Another study determined that miR-200b/c targeted RECK and subsequently upregulated Skp2 expression and inhibited p27 levels in colorectal cancer cells, resulting in enhanced cell proliferation [31]. Moreover, miR-3163 inhibited the expression of Skp2 in non-small cell lung cancer (NSCLC) cells and suppressed the growth of NSCLC cells [32]. In addition, miR-186 suppressed proliferation and stimulated apoptosis by targeting Skp2 in esophageal squamous cell carcinoma [33]. One group reported that miR-508-5p retarded metastasis by inhibition of Skp2 in gastric cancer cells [34]. Another group showed that miR-1297 increased cell proliferation by directly targeting PTEN and subsequently upregulating Skp2 in NSCLC cells [35]. In addition, miR-340 displayed antineoplastic functions via suppressing Skp2 in liver carcinoma [36]. Here, we reported that miR-506 targeted Skp2 and led to antitumor activity in osteosarcoma cells.

Accumulated evidence has indicated that miR-506 has a potential role in osteosarcoma cells [14, 15]. IL-1β activated nuclear factor kappa B (NF-κB) and subsequently decreased expression of miR-506, promoting growth of osteosarcoma cells by targeting Jagged-1 [37]. Another group reported that miR-506-3p attenuated proliferation and metastasis via inhibition of Ras-related protein Rab-3D expression in osteosarcoma cells [16]. Moreover, miR-506-3p induced mesenchymal-to-epithelial transition and inhibited autophagy by regulation of SPHK1 (sphingosine kinase 1) in osteosarcoma cells [38]. The long noncoding RNA RHPN1-AS1 was demonstrated to facilitate osteosarcoma progression by sponging miR-506 and activating Snail2 expression [39]. CircRNA UBAP2 increased the expression of Sema6D in osteosarcoma cells and led to cisplatin resistance via sponging miR-506-3p [40]. We clarified that miR-506 performed anticancer activity via suppression of Skp2 in osteosarcoma cells.

Our work presents the anticancer effect of miR-506 in osteosarcoma, and demonstrates that minimization of Skp2 by miR-506 might be a novel tactics for treating osteosarcoma. Further exploration is needed to ascertain whether miR-506 softens osteosarcoma tumor growth in mice. It is also necessary to measure whether miR-506 has a negative association with Skp2 expression in osteosarcoma tissues. Last, it is important to discover the safe miR-506 mimics for clinical trials in osteosarcoma patients in the future.

## MATERIALS AND METHODS

### Cell culture

The human osteosarcoma MG63 and U2OS cell lines were used to detect the effects of miR-506. These cells were grown in DMEM at 37° C in a 5% CO_2_ atmosphere.

### Transfection

Cells were seeded onto 96-well or six-well plates and incubated overnight. Then, osteosarcoma cells were transfected with miR-506 mimics, Skp2 cDNA, Skp2 siRNA or a combination of these reagents via Lipofectamine 2000.

### Cell viability

After MG63 cells and U2OS cells were transfected with miR-506 mimics by Lipofectamine 2000, the cells were incubated onto 96-well plates for different times. Then, MTT was added to the wells and the cells were cultured for 4 hours. Then, OD values were measured by the microplate reader.

### Cell apoptosis

Cells were incubated in six-well plates overnight and transfected with miR-506 mimics, Skp2 cDNA, Skp2 siRNA or a combination for 72 hours. Then the cells were collected and apoptosis was analyzed [41].

### Scratch wound healing

The treated cells were seeded onto 6-well plates and incubated with complete DMEM until confluence. After the cells were serum starved overnight, scratch wounds were generated by a yellow pipette tip. Cell debris was removed by washing twice. After 20 hours, images were captured by microscopy to illustrate wound areas.

### Cell invasion assay

The transfected cells were seeded into the Transwell upper chamber containing serum-free medium with the Matrigel-covered membrane. After twenty hours, the invaded cells were incubated with calcein AM. Invaded cells were imaged using a microscope.

### Real-time PCR

Total RNA was harvested using the Trizol reagent from transfected osteosarcoma cells. The cDNA was synthesized by reverse transcription using miRNA primers. All primers were purchased from GenePharma. PCR was carried out to examine the expression of miR-506 as described previously [42].

### Western blotting analysis

Treated cells were centrifuged and lysed by RIPA lysis buffer, and proteins in cell lysates were subjected to SDS-PAGE and further transferred to PVDF membrane. The western blotting was done as described before [41].

### Luciferase reporter activity assay

The target sequence of the Skp2 miRNA 3’-UTR clone was illustrated and cloned. Moreover, U2OS cells transfected with miR-506 mimics were seeded onto 24-well plates for 24 hours. Then, cells were transfected with 1 μg of luciferase reporter plasmids in each well. The dual-luciferase reporter assay kit was applied for measuring luciferase activities following the manufacturer’s protocol [43].

### Statistical analysis

The unpaired two-tailed Student’s t test was applied for comparing the differences between two data sets. One-way ANOVA was selected to evaluate multiple data sets. P < 0.05 was considered significant.

## References

[1] Sadykova LR, Ntekim AI, Muyangwa-Semenova M, Rutland CS, Jeyapalan JN, Blatt N, Rizvanov AA. Epidemiology and risk factors of osteosarcoma. Cancer Invest. 2020; 38:259–69. 10.1080/07357907.2020.1768401 32400205

[2] Zhang B, Zhang Y, Li R, Li J, Lu X, Zhang Y. The efficacy and safety comparison of first-line chemotherapeutic agents (high-dose methotrexate, doxorubicin, cisplatin, and ifosfamide) for osteosarcoma: a network meta-analysis. J Orthop Surg Res. 2020; 15:51. 10.1186/s13018-020-1576-0 32054494 PMC7020590

[3] Anderson PM. Radiopharmaceuticals for treatment of osteosarcoma. Adv Exp Med Biol. 2020; 1257:45–53. 10.1007/978-3-030-43032-0_4 32483729

[4] Bishop MW, Janeway KA, Gorlick R. Future directions in the treatment of osteosarcoma. Curr Opin Pediatr. 2016; 28:26–33. 10.1097/MOP.0000000000000298 26626558 PMC4761449

[5] Allison DC, Carney SC, Ahlmann ER, Hendifar A, Chawla S, Fedenko A, Angeles C, Menendez LR. A meta-analysis of osteosarcoma outcomes in the modern medical era. Sarcoma. 2012; 2012:704872. 10.1155/2012/704872 22550423 PMC3329715

[6] de Azevedo JW, de Medeiros Fernandes TA, Fernandes JV Jr, de Azevedo JC, Lanza DC, Bezerra CM, Andrade VS, de Araújo JM, Fernandes JV. Biology and pathogenesis of human osteosarcoma. Oncol Lett. 2020; 19:1099–116. 10.3892/ol.2019.11229 31966039 PMC6955653

[7] Otoukesh B, Abbasi M, Gorgani HO, Farahini H, Moghtadaei M, Boddouhi B, Kaghazian P, Hosseinzadeh S, Alaee A. MicroRNAs signatures, bioinformatics analysis of miRNAs, miRNA mimics and antagonists, and miRNA therapeutics in osteosarcoma. Cancer Cell Int. 2020; 20:254. 10.1186/s12935-020-01342-4 32565738 PMC7302353

[8] Wang J, Liu S, Shi J, Li J, Wang S, Liu H, Zhao S, Duan K, Pan X, Yi Z. The role of miRNA in the diagnosis, prognosis, and treatment of osteosarcoma. Cancer Biother Radiopharm. 2019; 34:605–13. 10.1089/cbr.2019.2939 31674804

[9] Li J, Ju J, Ni B, Wang H. The emerging role of miR-506 in cancer. Oncotarget. 2016; 7:62778–88. 10.18632/oncotarget.11294 27542202 PMC5308765

[10] Xia XY, Yu YJ, Ye F, Peng GY, Li YJ, Zhou XM. MicroRNA-506-3p inhibits proliferation and promotes apoptosis in ovarian cancer cell via targeting SIRT1/AKT/FOXO3a signaling pathway. Neoplasma. 2020; 67:344–53. 10.4149/neo_2020_190517N441 31973537

[11] Zhang L, Zhou H, Wei G. miR-506 regulates cell proliferation and apoptosis by affecting RhoA/ROCK signaling pathway in hepatocellular carcinoma cells. Int J Clin Exp Pathol. 2019; 12:1163–73. 31933931 PMC6947048

[12] Wang GJ, Jiao BP, Liu YJ, Li YR, Deng BB. Reactivation of microRNA-506 inhibits gastric carcinoma cell metastasis through ZEB2. Aging (Albany NY). 2019; 11:1821–31. 10.18632/aging.101877 30923258 PMC6461178

[13] Liang TS, Zheng YJ, Wang J, Zhao JY, Yang DK, Liu ZS. MicroRNA-506 inhibits tumor growth and metastasis in nasopharyngeal carcinoma through the inactivation of the Wnt/β-catenin signaling pathway by down-regulating LHX2. J Exp Clin Cancer Res. 2019; 38:97. 10.1186/s13046-019-1023-4 30791932 PMC6385449

[14] Yu Z, Zhang Y, Gao N, Wang X. Overexpression of miR-506 inhibits growth of osteosarcoma through Snail2. Am J Transl Res. 2015; 7:2716–23. 26885269 PMC4731669

[15] Yao J, Qin L, Miao S, Wang X, Wu X. Overexpression of miR-506 suppresses proliferation and promotes apoptosis of osteosarcoma cells by targeting astrocyte elevated gene-1. Oncol Lett. 2016; 12:1840–48. 10.3892/ol.2016.4827 27602115 PMC4998420

[16] Jiashi W, Chuang Q, Zhenjun Z, Guangbin W, Bin L, Ming H. MicroRNA-506-3p inhibits osteosarcoma cell proliferation and metastasis by suppressing RAB3D expression. Aging (Albany NY). 2018; 10:1294–305. 10.18632/aging.101468 29905536 PMC6046236

[17] Asmamaw MD, Liu Y, Zheng YC, Shi XJ, Liu HM. Skp2 in the ubiquitin-proteasome system: a comprehensive review. Med Res Rev. 2020; 40:1920–49. 10.1002/med.21675 32391596

[18] Yan L, Lin M, Pan S, Assaraf YG, Wang ZW, Zhu X. Emerging roles of F-box proteins in cancer drug resistance. Drug Resist Updat. 2020; 49:100673. 10.1016/j.drup.2019.100673 31877405

[19] Cai Z, Moten A, Peng D, Hsu CC, Pan BS, Manne R, Li HY, Lin HK. The Skp2 pathway: a critical target for cancer therapy. Semin Cancer Biol. 2020; 67:16–33. 10.1016/j.semcancer.2020.01.013 32014608 PMC9201937

[20] Wang Z, Fukushima H, Inuzuka H, Wan L, Liu P, Gao D, Sarkar FH, Wei W. Skp2 is a promising therapeutic target in breast cancer. Front Oncol. 2012; 1:57. 10.3389/fonc.2011.00057 22279619 PMC3263529

[21] Wang Z, Gao D, Fukushima H, Inuzuka H, Liu P, Wan L, Sarkar FH, Wei W. Skp2: a novel potential therapeutic target for prostate cancer. Biochim Biophys Acta. 2012; 1825:11–17. 10.1016/j.bbcan.2011.09.002 21963805 PMC3242930

[22] Wang Z, Liu P, Inuzuka H, Wei W. Roles of f-box proteins in cancer. Nat Rev Cancer. 2014; 14:233–47. 10.1038/nrc3700 24658274 PMC4306233

[23] Li Z, Liu H, Li B, Zhang Y, Piao C. Saurolactam inhibits proliferation, migration, and invasion of human osteosarcoma cells. Cell Biochem Biophys. 2015; 72:719–26. 10.1007/s12013-015-0523-x 25627547

[24] Chen X, Li Q, He Y, Du H, Zhan Z, Zhao H, Shi J, Ye Q, Hu J. 15,16-dihydrotanshinone I induces apoptosis and inhibits the proliferation, migration of human osteosarcoma cell line 143B *in vitro*. Anticancer Agents Med Chem. 2017; 17:1234–42. 10.2174/1871520615666151019092919 26478521

[25] Ding L, Li R, Han X, Zhou Y, Zhang H, Cui Y, Wang W, Bai J. Inhibition of Skp2 suppresses the proliferation and invasion of osteosarcoma cells. Oncol Rep. 2017; 38:933–40. 10.3892/or.2017.5713 28627672

[26] Ding L, Li R, Sun R, Zhou Y, Zhou Y, Han X, Cui Y, Wang W, Lv Q, Bai J. S-phase kinase-associated protein 2 promotes cell growth and motility in osteosarcoma cells. Cell Cycle. 2017; 16:1547–55. 10.1080/15384101.2017.1346760 28771075 PMC5584850

[27] Zhang Y, Zvi YS, Batko B, Zaphiros N, O’Donnell EF, Wang J, Sato K, Yang R, Geller DS, Koirala P, Zhang W, Du X, Piperdi S, et al. Down-regulation of Skp2 expression inhibits invasion and lung metastasis in osteosarcoma. Sci Rep. 2018; 8:14294. 10.1038/s41598-018-32428-9 30250282 PMC6155331

[28] Ding L, Wang C, Cui Y, Han X, Zhou Y, Bai J, Li R. S-phase kinase-associated protein 2 is involved in epithelial-mesenchymal transition in methotrexate-resistant osteosarcoma cells. Int J Oncol. 2018; 52:1841–52. 10.3892/ijo.2018.4345 29620168 PMC5919717

[29] Sanchez N, Gallagher M, Lao N, Gallagher C, Clarke C, Doolan P, Aherne S, Blanco A, Meleady P, Clynes M, Barron N. MiR-7 triggers cell cycle arrest at the G1/S transition by targeting multiple genes including Skp2 and Psme3. PLoS One. 2013; 8:e65671. 10.1371/journal.pone.0065671 23762407 PMC3675065

[30] Qi F, He T, Jia L, Song N, Guo L, Ma X, Wang C, Xu M, Fu Y, Li L, Luo Y. The miR-30 family inhibits pulmonary vascular hyperpermeability in the premetastatic phase by direct targeting of Skp2. Clin Cancer Res. 2015; 21:3071–80. 10.1158/1078-0432.CCR-14-2785 25810374

[31] Pan Y, Liang H, Chen W, Zhang H, Wang N, Wang F, Zhang S, Liu Y, Zhao C, Yan X, Zhang J, Zhang CY, Gu H, et al. microRNA-200b and microRNA-200c promote colorectal cancer cell proliferation via targeting the reversion-inducing cysteine-rich protein with kazal motifs. RNA Biol. 2015; 12:276–89. 10.1080/15476286.2015.1017208 25826661 PMC4615722

[32] Su L, Han D, Wu J, Huo X. Skp2 regulates non-small cell lung cancer cell growth by Meg3 and miR-3163. Tumour Biol. 2016; 37:3925–31. 10.1007/s13277-015-4151-2 26482610

[33] He W, Feng J, Zhang Y, Wang Y, Zang W, Zhao G. microRNA-186 inhibits cell proliferation and induces apoptosis in human esophageal squamous cell carcinoma by targeting SKP2. Lab Invest. 2016; 96:317–24. 10.1038/labinvest.2015.134 26568291

[34] Duan X, Bai J, Wei J, Li Z, Liu X, Xu G. MicroRNA-508-5p suppresses metastasis in human gastric cancer by targeting S-phase kinase-associated protein 2. Mol Med Rep. 2017; 16:2163–71. 10.3892/mmr.2017.6793 28627698

[35] Bu W, Luo T. miR-1297 promotes cell proliferation of non-small cell lung cancer cells: involving in PTEN/Akt/Skp2 signaling pathway. DNA Cell Biol. 2017; 36:976–82. 10.1089/dna.2017.3886 28872922

[36] Wang Z, Song D, Huang P. MicroRNA-340 inhibits tumor cell proliferation, migration and invasion, and induces apoptosis in hepatocellular carcinoma. Mol Med Rep. 2017; 16:7649–56. 10.3892/mmr.2017.7583 28944918

[37] Hu M, Yuan X, Liu Y, Tang S, Miao J, Zhou Q, Chen S. IL-1β-induced NF-κB activation down-regulates miR-506 expression to promotes osteosarcoma cell growth through JAG1. Biomed Pharmacother. 2017; 95:1147–55. 10.1016/j.biopha.2017.08.120 28926924

[38] Wang D, Bao F, Teng Y, Li Q, Li J. MicroRNA-506-3p initiates mesenchymal-to-epithelial transition and suppresses autophagy in osteosarcoma cells by directly targeting SPHK1. Biosci Biotechnol Biochem. 2019; 83:836–44. 10.1080/09168451.2019.1569496 30669957

[39] Wang L, Liu Y. Long noncoding RNA RHPN1-AS1 exerts pro-oncogenic actions in osteosarcoma by functioning as a molecular sponge of miR-506 to positively regulate SNAI2 expression. Cell Cycle. 2020; 19:1517–29. 10.1080/15384101.2020.1762039 32401134 PMC7469572

[40] Gong X, Li W, Dong L, Qu F. CircUBAP2 promotes SEMA6D expression to enhance the cisplatin resistance in osteosarcoma through sponging miR-506-3p by activating Wnt/β-catenin signaling pathway. J Mol Histol. 2020; 51:329–40. 10.1007/s10735-020-09883-8 32472335 PMC7368871

[41] Li R, Yan Q, Tian P, Wang Y, Wang J, Tao N, Ning L, Lin X, Ding L, Liu J, Ma C. CBX7 inhibits cell growth and motility and induces apoptosis in cervical cancer cells. Mol Ther Oncolytics. 2019; 15:108–16. 10.1016/j.omto.2019.09.002 31709304 PMC6834976

[42] Xia J, Cao T, Ma C, Shi Y, Sun Y, Wang ZP, Ma J. miR-7 suppresses tumor progression by directly targeting MAP3K9 in pancreatic cancer. Mol Ther Nucleic Acids. 2018; 13:121–32. 10.1016/j.omtn.2018.08.012 30290304 PMC6171162

[43] Ma J, Cao T, Cui Y, Zhang F, Shi Y, Xia J, Wang ZP. miR-223 regulates cell proliferation and invasion via targeting PDS5B in pancreatic cancer cells. Mol Ther Nucleic Acids. 2019; 14:583–92. 10.1016/j.omtn.2019.01.009 30776580 PMC6378631

